# Proposal for the use of echocardiography in bloodstream infections due to different streptococcal species

**DOI:** 10.1186/s12879-021-06391-2

**Published:** 2021-07-16

**Authors:** Sandra Chamat-Hedemand, Niels Eske Bruun, Lauge Østergaard, Magnus Arpi, Emil Fosbøl, Jonas Boel, Louise Bruun Oestergaard, Trine K. Lauridsen, Gunnar Gislason, Christian Torp-Pedersen, Anders Dahl

**Affiliations:** 1 Department of Cardiology, Zealand University Hospital, Roskilde, Denmark; 2 Department of Cardiology, Copenhagen University Hospital Herlev and Gentofte, Hellerup, Denmark; 3 Institute of Clinical Medicine, Copenhagen University, Copenhagen, Denmark; 4 Clinical Institute, Aalborg University, Aalborg, Denmark; 5 Department of Cardiology, The Heart Centre, Copenhagen University Hospital Rigshospitalet, Copenhagen Ø, Denmark; 6 Department of Clinical Microbiology, Copenhagen University Hospital Herlev and Gentofte, Herlev, Denmark; 7 The Danish Heart Foundation, Copenhagen, Denmark; 8 Department of Cardiology and Clinical Research, Nordsjaellands Hospital, Hillerød, Denmark; 9 Department of Cardiology, Aalborg University Hospital, Aalborg, Denmark

**Keywords:** Infective endocarditis, Streptococcal species, Bloodstream infection, Echocardiography

## Abstract

**Background:**

Infective endocarditis (IE) is diagnosed in 7–8% of streptococcal bloodstream infections (BSIs), yet it is unclear when to perform transthoracic (TTE) and transoesophageal echocardiography (TOE) according to different streptococcal species. The aim of this sub-study was to propose a flowchart for the use of echocardiography in streptococcal BSIs.

**Methods:**

In a population-based setup, we investigated all patients admitted with streptococcal BSIs and crosslinked data with nationwide registries to identify comorbidities and concomitant hospitalization with IE. Streptococcal species were divided in four groups based on the crude risk of being diagnosed with IE (low-risk < 3%, moderate-risk 3–10%, high-risk 10–30% and very high-risk > 30%). Based on number of positive blood culture (BC) bottles and IE risk factors (prosthetic valve, previous IE, native valve disease, and cardiac device), we further stratified cases according to probability of concomitant IE diagnosis to create a flowchart suggesting TTE plus TOE (IE > 10%), TTE (IE 3–10%), or “wait & see” (IE < 3%).

**Results:**

We included 6393 cases with streptococcal BSIs (mean age 68.1 years [SD 16.2], 52.8% men). BSIs with low-risk streptococci (*S. pneumoniae, S. pyogenes, S. intermedius*) are not initially recommended echocardiography, unless they have ≥3 positive BC bottles and an IE risk factor. Moderate-risk streptococci (*S. agalactiae, S. anginosus, S. constellatus, S. dysgalactiae, S. salivarius, S. thermophilus*) are guided to “wait & see” strategy if they neither have a risk factor nor ≥3 positive BC bottles, while a TTE is recommended if they have either ≥3 positive BC bottles or a risk factor. Further, a TTE and TOE are recommended if they present with both. High-risk streptococci (*S. mitis/oralis, S. parasanguinis*, *G. adiacens*) are directed to a TTE if they neither have a risk factor nor ≥3 positive BC bottles, but to TTE and TOE if they have either ≥3 positive BC bottles or a risk factor. Very high-risk streptococci (*S. gordonii, S. gallolyticus, S. mutans, S. sanguinis*) are guided directly to TTE and TOE due to a high baseline IE prevalence.

**Conclusion:**

In addition to the clinical picture, this flowchart based on streptococcal species, number of positive blood culture bottles, and risk factors, can help guide the use of echocardiography in streptococcal bloodstream infections. Since echocardiography results are not available the findings should be confirmed prospectively with the use of systematic echocardiography.

**Supplementary Information:**

The online version contains supplementary material available at 10.1186/s12879-021-06391-2.

## Background

Streptococci are one of the leading causes of infective endocarditis (IE), yet there is uncertainty when to screen for IE in patients with streptococcal bloodstream infections (BSIs) [1–4]. A nationwide registry study found an IE prevalence of 16.7% in *Enterococcus faecalis (E. faecalis)*, 10.1% in *Staphylococcus aureus (S. aureus),* and 7.3% in streptococcal bloodstream infections (BSIs), leading the authors to suggest that screening for IE in these patients seems reasonable [5]. In both *S. aureus* and *E. faecalis* BSIs a more extensive use of echocardiography has been recommended based on echocardiographic screening studies finding a IE prevalence around 15–25% [6–8].

In a recent large cohort study, we showed that different streptococcal species had markedly different IE prevalence, ranging from 1 to 2% in *S. pneumoniae* and *S. pyogenes* to almost 50% in *S. gordonii* and *S. mutans* [9]. These findings support the fact that not all streptococcal species carry the same risk of IE and therefore it is highly relevant to consider if the work-up should be differentiated between patients infected with different streptococcal species. The current European and American guidelines on IE are not specifying anything regarding the prevalence of IE nor the differentiation of work-up in patients with BSIs due to different streptococcal species [10, 11]. Furthermore, risk factors for IE in patients with a streptococcal BSI have been sparsely investigated. Sunnerhagen et al. created a score to guide the use of echocardiography in non-β-haemolytic streptococcal BSIs based on different risk factors [12]. However, the study was limited by low numbers, not differentiating between when to perform transthoracic (TTE) or transoesophageal echocardiography (TOE) and failing to include all the different streptococcal species.

In the present study our aim was to propose a flowchart for the use of echocardiography in patients with streptococcal BSIs based on retrospective data of IE prevalence according to streptococcal species and IE risk factors.

## Methods

### Study population, data sources and case definitions

This is a sub-study to a population-based study including adult patients with monospecies streptococcal BSIs admitted from January 1, 2008 to December 31, 2017 in the Capital Region of Denmark (1.5 million people) [9]. The full method has been described in detail in the main study. In addition to the main study exclusion of cases with unavailable species identification, the current sub-study also excluded rare species accounting for < 0.5% of BSIs. Using Danish nationwide registries [13, 14] we identified IE cases with hospital admissions with primary and secondary ICD-10 codes (I33, I38 and I39.8) and IE was considered associated to the streptococcal BSI, if the positive blood cultures occurred during the IE admission or up to 30 days prior. Since detailed patient chart data including data on echocardiograms are not available in the registries the estimated IE prevalence is based on the assumption that when the hospital doctors diagnose a patient with IE the patient actually has IE and in the same way, when a patient is not diagnosed with IE it is because the patient does not have IE. To reduce the number of false positive IE diagnoses, we required IE cases to have a hospital admission of minimum 14 days unless they died during the first 14 days of admission because this algorithm have been validated with a positive predictive value (PPV) above 90% for the diagnosis of IE in the Danish registries [15, 16]. See Supplemental Table 1 for ICD-, procedure- and ATC-codes used in this study.

### Design of echocardiography flowchart

Based on the crude estimated IE prevalence, each streptococcal species was assigned to a risk group: low (< 3%), moderate (3–10%), high (10–30%), and very high (> 30%). After grouping the streptococcal species in risk groups, the IE prevalence was further assessed in subgroups according to number of positive blood culture bottles and presence of conventional IE risk factors (prosthetic valve, previous IE, native valve disease and cardiac device) [17, 18]. From these subgroups we decided to use the following cut-offs for the proposed use of TTE and TOE: (i) IE prevalence > 10%, TTE and are recommended (ii) IE prevalence of 3–10%, TTE is recommended (iii) IE prevalence < 3%, “wait & see” is recommended, meaning that no initial echocardiography is recommended unless there is a strong clinical suspicion of IE.

### Statistics

Baseline data were presented for the four streptococcal risk groups. Categorical data were expressed as numbers and percentage (%), while continuous variables were presented as mean ± standard deviation (SD). Estimated prevalence of IE was calculated as BSI cases with concomitant IE diagnosis divided by the total number of BSI cases in the total cohort and in the different streptococcal species groups, respectively. The IE prevalence estimate was presented with a 95% confidence interval (CI). A multivariable logistic regression analysis with the outcome IE diagnosis in BSIs with different streptococcal species, adjusted for age, sex, ≥3 positive blood culture bottles, native valve disease, prosthetic valve, previous IE, and cardiac device, was carried out with *S. pneumoniae* as a reference species. Results were presented as odds ratio (OR) with 95% CI. The IE prevalence estimate of every step in the flowchart was calculated as cases with an IE diagnosis divided by the total number of BSI cases in each subgroup, with < or ≥ 3 positive blood culture bottles, and presence vs. absence of an IE risk factor. IE prevalence estimates were presented with a 95% confidence interval (CI). All statistical analyses were performed using the SAS statistical software, version 9.4 (SAS Institute, Inc., Cary, NC, USA).

## Results

### Patient inclusion and characteristics

**Fig. 1:**
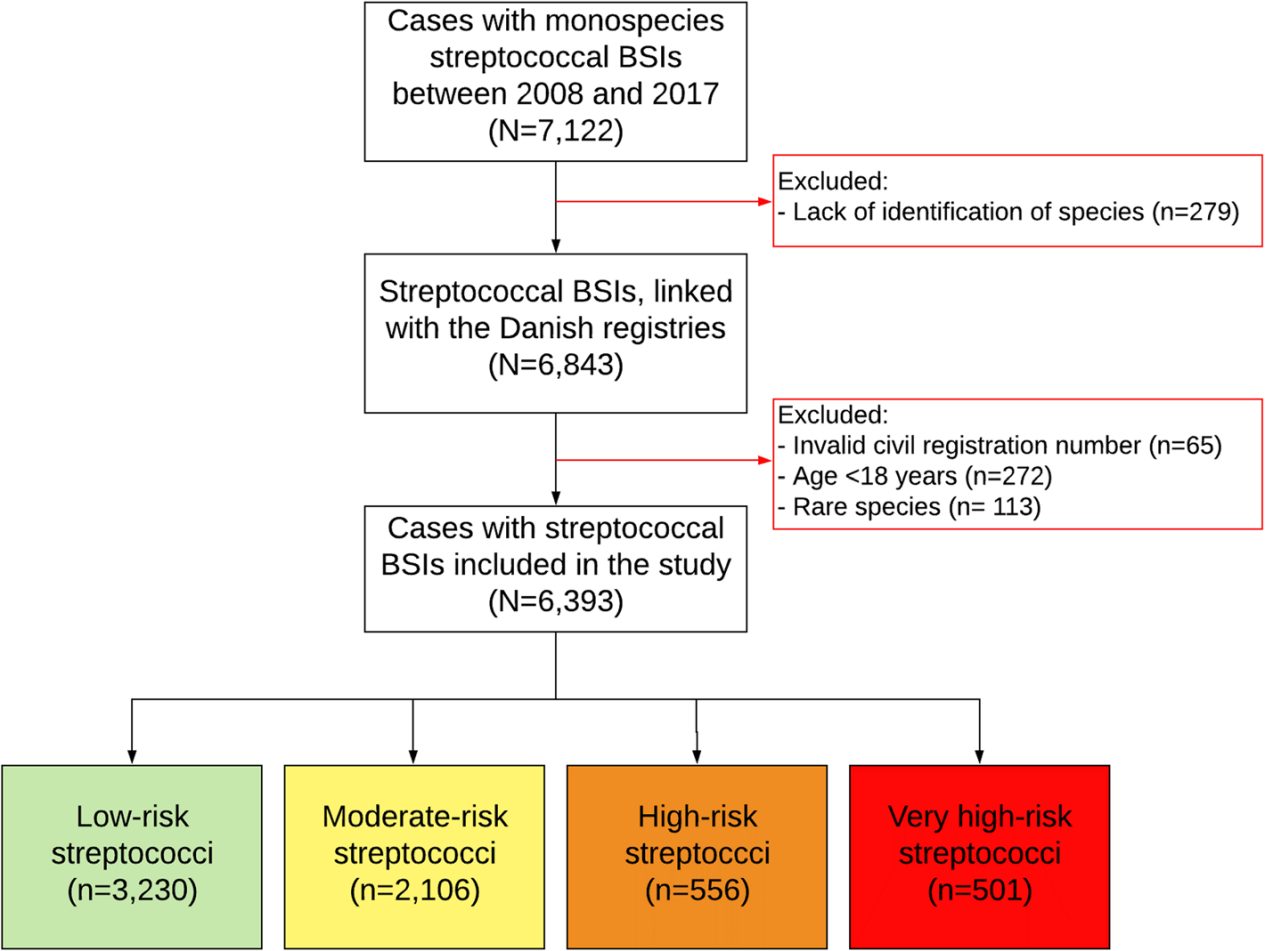
Patient inclusion. The figure illustrates a flowchart of patient selection. A total of 7122 cases of monospecies streptococcal BSIs were identified. Classification of species identification was unavailable in 279 cases. From the remaining 6843 cases, 65 cases had an invalid civil registration number, 272 cases were below 18 years of age, and 113 cases were infected with rare streptococcal species. BSI, bloodstream infection. Green: low-risk species (IE prevalence < 3%), yellow: moderate-risk species (IE prevalence 3–10%), orange: high-risk species (IE prevalence 10–30%), red: very high-risk species (IE prevalence > 30%)

From a total of 7122 streptococcal BSI cases we identified 6506 eligible cases with monospecies streptococcal BSIs in 6224 unique patients, of which 236 patients had more than one BSI case with a median of 332 days [IQR 102–869] between the first and the second episode. After excluding rare species (causing < 0.5% of BSIs) we included 6393 cases with monospecies streptococcal BSIs of which 451 were diagnosed with IE (7.1%) (Fig. 1). Based on the IE prevalence streptococcal species were classified as low-risk, moderate-risk, high-risk and very high-risk streptococci as described in the main study [9]. There were 3230 BSI cases (51%) in the low-risk group, 2106 BSI cases (33%) in the moderate-risk group, 556 BSI cases (9%) in the high-risk group, and 501 BSI cases (8%) in the very high-risk group (Fig. 1). In the total cohort the mean age was 68.1 years (SD 16.2) and 52.8% were men. Baseline characteristics according to risk groups are shown in Table 1. Cases in the very high-risk streptococci group were older and had the highest rate of native valve disease (13.2%), prosthetic valve (12.4%), previous IE (4.4%), and cardiac device (9.2%) (Table 1).

**Table 1:** Baseline characteristics

	Low-risk Streptococci (***n*** = 3230)	Moderate-risk streptococci (***n*** = 2106)	High-risk streptococci (***n*** = 556)	Very high-risk streptococci (***n*** = 501)	All BSIs (***N*** = 6393)
Demographics					
Age, mean (SD)	66.8 (16.5)	69.4 (15.6)	67.0 (17.1)	72.2 (14.2)	68.1 (16.2)
Male, n (%)	1543 (47.8)	1204 (57.2)	325 (58.5)	301 (60.1)	3373 (52.8)
Comorbidities ^a^					
Native valve disease ^b^, n (%)	97 (3.0)	106 (5.0)	38 (6.8)	66 (13.2)	307 (4.8)
Prosthetic valve, n (%)	47 (1.5)	61 (2.9)	28 (5.0)	62 (12.4)	198 (3.1)
Previous IE, n (%)	23 (0.7)	34 (1.6)	12 (2.2)	22 (4.4)	91 (1.4)
Cardiac device ^c^, n (%)	120 (3.7)	101 (4.8)	26 (4.7)	46 (9.2)	293 (4.6)
IHD, n (%)	478 (14.8)	428 (20.3)	128 (23.0)	138 (27.5)	1172 (18.3)
CHF, n (%)	365 (11.3)	347 (16.5)	89 (16.0)	123 (24.6)	924 (14.5)
Cancer, n (%)	787 (24.4)	671 (31.9)	162 (29.1)	130 (25.9)	1750 (27.4)
COPD, n (%)	612 (18.9)	280 (13.3)	105 (18.9)	86 (17.2)	1083 (16.9)
DM, n (%)	386 (12.0)	372 (17.7)	84 (15.1)	101 (20.2)	943 (14.8)
Renal disease, n (%)	230 (7.1)	209 (9.9)	64 (11.5)	57 (11.4)	560 (8.8)
Renal dialysis, n (%)	44 (1.4)	40 (1.9)	13 (2.3)	15 (3.0)	112 (1.8)

*BSI* Bloodstream infection, *CHF* Congestive heart failure, *COPD* Chronic obstructive pulmonary disease, *DM* Diabetes mellitus, *IE* Infective endocarditis, *IHD* Ischemic heart disease

^a^ medical history prior to IE, ^b^ Native valve disease without prosthetic valve, ^c^ cardiac implantable electronic device

### Adjusted risk of infective endocarditis

**Fig. 2:**
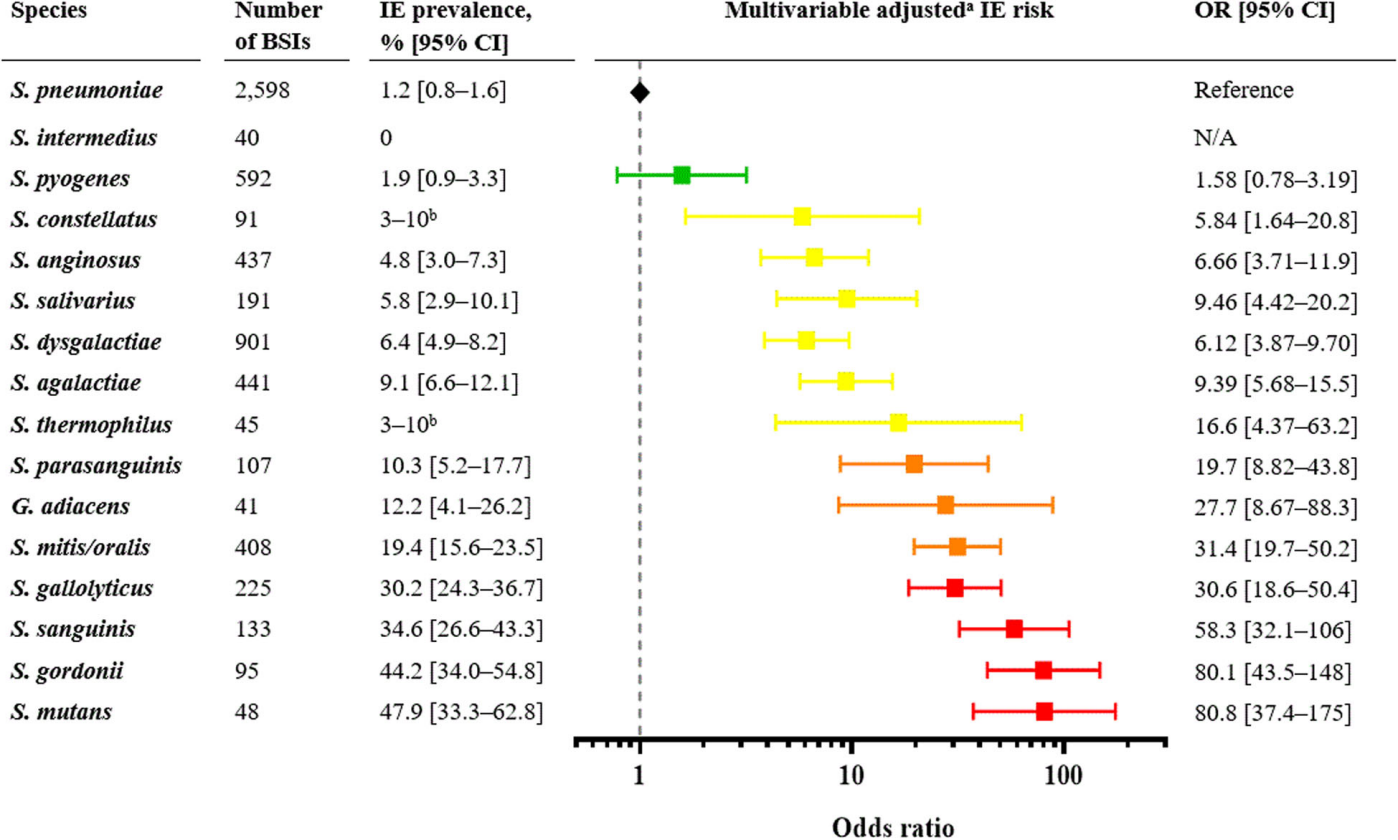
Prevalence and adjusted risk of IE. The figure illustrates the IE prevalence in different streptococcal species. In addition, a multivariable logistic regression analysis of the IE risk, adjusted for age, sex, ≥3 positive blood culture bottles, and risk factors, is presented. S. pneumoniae BSI is set as a reference. The results are presented as OR (95% CI). The figure is partly adapted from the main study [9]. .^a^ adjusted for age, sex, ≥3 positive blood culture bottles, native valve disease, prosthetic valve, previous IE, and cardiac device. ^b^ The exact number cannot be presented due to microdata. BSI, bloodstream infection; CI confidence interval; IE, infective endocarditis; OR, odds ratio. Green: low-risk species (IE prevalence < 3%), yellow: moderate-risk species (IE prevalence 3–10%), orange: high-risk species (IE prevalence 10–30%), red: very high-risk species (IE prevalence > 30%)

Figure 2 shows the crude prevalence of IE according to streptococcal species and the multivariable logistic regression adjusted for age, sex, and IE risk factors (≥ 3 positive blood culture bottles, native valve disease, prosthetic valve, previous IE and cardiac device). In the adjusted analysis including a slightly smaller cohort than in the main study, the overall risk pattern remained stable with progressively higher associated risk of IE in the species with the higher IE prevalence (Fig. 2).

### Endocarditis prevalence according to species, number of positive blood culture bottles, and risk factors

The IE prevalence was calculated for each streptococcal risk group and is presented relative to the number of positive blood culture bottles, and IE risk factors (Table 2). Low-risk streptococci (*S. pneumoniae, S. pyogenes,* and *S. intermedius*) had an IE prevalence of 0.5–1.5%, unless they had ≥3 positive blood culture bottles and at least one further risk factor in which case IE prevalence was 6.9% [95% CI: 3.4–12.3%]. Moderate-risk streptococci (*S. agalactiae, S. anginosus, S. constellatus, S. dysgalactiae, S. salivarius,* and *S. thermophilus*) had an IE prevalence of 1.5% [95% CI: 0.9–2.4%] if they neither had a risk factor nor ≥3 positive blood culture bottles, but an IE prevalence of 3–10% if they had either ≥3 positive blood culture bottles (IE prevalence 9.6% [95% CI: 7.5–12.1%]) or a risk factor (IE prevalence 8.7% [95% CI: 4.4–15.1%]). Moderate-risk streptococci presenting with both ≥3 positive blood culture bottles and a risk factor had an IE prevalence of 31.1% [95% CI: 23.3–39.7%]. High-risk streptococci (*S. mitis/oralis, S. parasanguinis*, and *G. adiacens*) had an IE prevalence of 3.6% [95% CI: 1.9–6.0%]) if they neither had a risk factor nor ≥3 positive blood culture bottles, but an IE prevalence of 14.0% [95% CI: 5.3–27.9%] if they presented a risk factor and < 3 positive blood culture bottles and an IE prevalence of 48.4% [95% CI: 40.4–56.5%] if they had ≥3 positive blood culture bottles. Finally, very high-risk streptococci (*S. gordonii, S. gallolyticus (formerly S. bovis), S. mutans,* and *S. sanguinis*) had a very high baseline IE prevalence of 35.7% [95% CI: 31.5–40.1%] (Table 2).

**Table 2:** IE prevalence for steps in the flowchart

	Number of BSIs	Number of IE cases	IE prevalence with [95% CI]
Low-risk species, baseline	3230	41	1.3% [0.9–1.7%]
< 3 positive BC bottles	1500	7	0.5% [0.2–1.0%]
≥ 3 positive BC bottles without risk factors	1585	24	1.5% [1.0–2.2%]
≥ 3 positive BC bottles with a risk factor	145	10	6.9% [3.4–12.3%]
Moderate-risk species, baseline	2106	136	6.5% [5.4–7.6%]
< 3 positive BC bottles without risk factors	1163	18	1.5% [0.9–2.4%]
< 3 positive BC bottles with a risk factor	126	11	8.7% [4.4–15.1%]
≥ 3 positive BC bottles without risk factors	685	66	9.6% [7.5–12.1%]
≥ 3 positive BC bottles with a risk factor	132	41	31.1% [23.3–39.7%]
High-risk species, baseline	556	95	17.1% [14.0–20.5%]
< 3 positive BC bottles without risk factors	356	13	3.6% [1.9–6.0%]
< 3 positive BC bottles with a risk factor	43	6	14.0% [5.3–27.9%]
≥ 3 positive BC bottles	157	76	48.4% [40.4–56.5%]
Very high-risk species, baseline	501	179	35.7% [31.5–40.1%]

*BC* Blood culture, *BSI* Bloodstream infection, *CI* Confidence interval, *IE* Infective endocarditis

Risk factors: native valve disease, prosthetic valve, previous IE or cardiac device

### Proposed flowchart for echocardiography in streptococcal bacteraemia

**Fig. 3:**
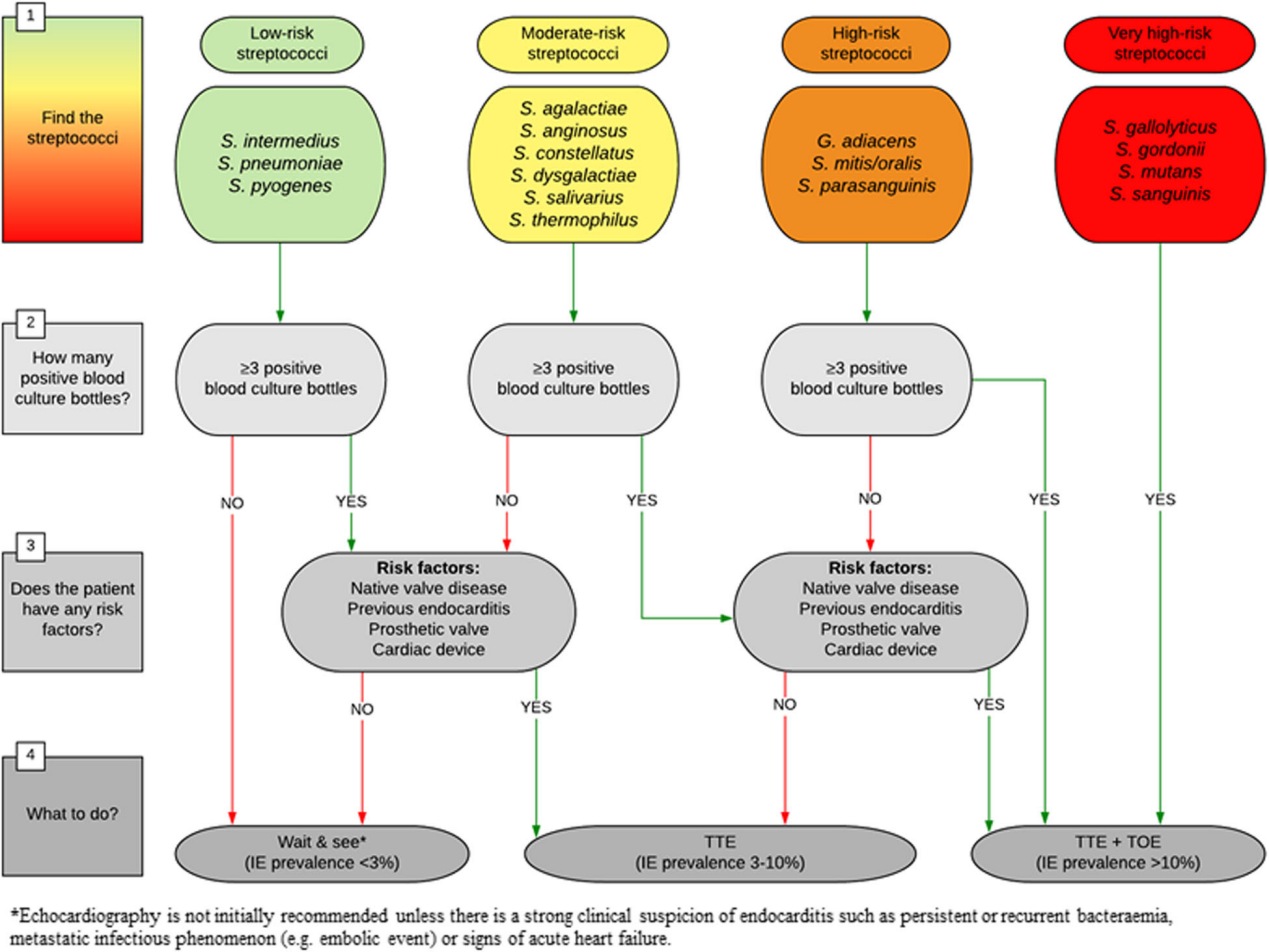
Echocardiography of streptococcal bloodstream infections. The figure shows a flowchart for the proposed use of echocardiography in patients with streptococcal bloodstream infections, based on streptococcal species, number of positive blood culture bottles, and presence of a risk factor. IE, infective endocarditis; TOE, transoesophageal echocardiography; TTE, transthoracic echocardiography

Based on the estimated IE prevalence in BSIs with different streptococcal species and taking the number of positive blood culture bottles and IE risk factors into account we designed a flowchart for the use of TTE and TOE (Fig. 3). Using the specified IE prevalence cut-offs, we propose the following strategy: “wait & see” (IE prevalence < 3%), TTE (IE prevalence 3–10%), and TTE plus TOE (IE prevalence > 10%). In this way, low-risk streptococci are directed to “wait & see”, unless they have ≥3 positive blood culture bottles and an IE risk factor in which case they are led to TTE. Moderate-risk streptococci with < 3 positive blood culture bottles and no risk factors are guided to “wait & see”, whereas a TTE is recommended if they have either ≥3 positive blood culture bottles or a risk factor. However, moderate-risk streptococci presenting with both ≥3 positive blood culture bottles and a risk factor are recommended a TTE plus TOE. High-risk streptococci are recommended a TTE if they neither have a risk factor nor ≥3 positive blood culture bottles, but a TTE plus TOE if they have either ≥3 positive blood culture bottles or a risk factor. Finally, very high-risk streptococci are recommended a TTE plus TOE due to a very high baseline IE prevalence.

## Discussion

Based on estimated IE prevalence, this study proposes a flowchart for the use of echocardiography in patients with BSIs caused by different streptococcal species. The main findings were: (i) BSI cases with a low-risk streptococcus (*S. pneumoniae, S. pyogenes* or *S. intermedius*) are not initially being recommended an echocardiography, unless they have ≥3 positive blood culture bottles and presence of an IE risk factor (ii) BSI cases with a very high-risk streptococcus (*S. gordonii, S. gallolyticus, S. mutans,* and *S. sanguinis*) or cases with ≥3 positive blood culture bottles with a high-risk streptococcus (*S. mitis/oralis, S. parasanguinis* or *G. adiacens*) are recommended to a TTE plus TOE.

To evaluate the possible clinical implications of these findings it is relevant to discuss the current clinical guidelines deciding when to perform TTE and/or TOE in patients with streptococcal BSIs. Neither IE guidelines from the European Society of Cardiology (ESC) nor the American Heart Association (AHA), are specifying anything regarding differentiation of work-up in patients with BSI due to different streptococcal species [10, 11]. However, in the diagnostic criteria for IE, which are based on the modified Duke criteria, BSIs with viridans streptococci and *S. bovis* are major diagnostic criteria [10, 19]. It is concerning that the term viridans streptococci covers all species in the *S. anginosus* group, *S. mitis* group, *S. mutans* group, and *S. salivarius* group without any distinction between specific species [20]. It is now evident, that different streptococcal species within the so called viridans streptococci have different IE prevalence, ranging from 4.8% in *S. anginosus* to 47.9% in *S. mutans* [9]. Therefore, it is highly relevant to differentiate the work-up in patients with streptococcal BSIs with different streptococcal species based on the associated risk of IE. To confront this problem, Sunnerhagen et al. created the HANDOC score based on 339 non-β-haemolytic streptococcal BSI cases and 26 cases of IE [12]. The score is aimed to guide the use of echocardiography by evaluating six factors: Heart murmur or valvular disease, Aetiology, Number of cultures, Duration of symptoms, Only one species, and Community acquisition. Despite the fact, that the score showed a high sensitivity in an external validation [21], the study was limited by only including few IE cases, not including all the different streptococcal species and not differentiating between when to perform TTE or TOE. Surprisingly, the authors only found 4 cases of IE in 102 *S. mitis* group BSIs in sharp contrast to most other studies finding *S. mitis* to cause the majority of IE cases in streptococcal BSIs [9, 22, 23]. Further, the authors suggest a reduction in the calculated score when patients are infected with *S. anginosus* BSIs, since they did not find any IE cases in 105 BSIs with *S. anginosus* [12]. This finding is not in line with recent findings in a much larger cohort, where the IE prevalence for *S. anginosus* BSIs was almost 5% (21 of 431) [9].

With nearly 6400 streptococcal BSI cases, we had sufficient numbers to evaluate the prevalence of IE on species level and stratify the species according to the prevalence of IE into low, moderate, high, and very high risk of IE. The overall estimated IE prevalence in our cohort (7.1%) was not markedly different from the one found in non-β-haemolytic streptococcal BSI (7.7%) and mixed streptococcal BSI (10.6%) [12, 24]. In addition, using the information from earlier studies of IE risk factors such as native valve disease, prosthetic valve, previous IE, and cardiac device we were able to incorporate these details in the flowchart [17, 18]. To determine the IE prevalence cut-offs for our proposed use of echocardiography we combined clinical experience with knowledge from earlier studies on typical IE bacteria to reach a consensus decision. Previous studies on *S. aureus* and *E. faecalis* IE have recommended TTE and TOE based on IE prevalence from 10 to 25% [5–7]. We decided to use an IE prevalence of 10% as cut-off for TOE to accommodate the fact that our numbers are likely to be conservative estimates lacking information on BSI cases where no echocardiography was performed (data not available). To select the IE prevalence limit for an expectant strategy (“wait & see”) we considered the prevalence of IE in *S. aureus* and *E. faecalis* BSI cases without any risk factors (3.4–5%) from earlier studies [6, 7]. Since these patients in low to moderate risk of IE are often still recommended a TTE we chose a lower limit of < 3% for the expectant strategy. In our study the IE prevalence in the subgroups of BSI cases leading to a “wait & see” strategy was well below this limit. It is adamant to underscore that the flowchart is thought as an additional tool to help the clinician and that the overall clinical assessment of the patient is still crucial. Therefore, clinical findings such as persistent or recurrent bacteraemia, signs of metastatic infection (e.g. embolic event) or acute heart failure should of course lead to echocardiography and work up for IE no matter the outcome of the flowchart.

### Limitations

The flowchart is created on retrospective data from a partly register-based setup, which naturally introduces limitations. Firstly, the diagnosis of IE was based on ICD-10 discharge codes with the inherent risk of misclassification bias. However, the applied method using ICD-10 codes in the Danish registries has been validated with a positive predictive value for the diagnosis of IE of 90% [15]. Since the negative predictive value of the IE diagnosis in the Danish registries has not been investigated, we cannot accurately estimate the amount of overlooked IE patients that were misclassified (false negatives) by not receiving an ICD-10 IE diagnosis. Secondly, since data on TTE and TOE are not available, the flowchart was created retrospectively without knowledge of the use of echocardiography in the different groups. Since echocardiography plays a central role in diagnosing IE it would have been relevant to know if some species were less often examined with echocardiography thereby increasing the probability of ascertainment bias and missed IE cases. Having that in mind, the IE prevalence should be interpreted as conservative estimates. At the same time, the missing data on echocardiograms introduces a risk of circular deduction where the species more typically examined by echocardiography also becomes the species where IE is diagnosed most often. In other words that the current clinical selection of patients for echocardiography are already performed in a manner parallel to the suggested flow chart without collective consciousness of the specific selection process. In this way the suggested flowchart may just be the first actual description of the performed clinical practice applied by IE experts. The only way to answer this question is to perform a prospective screening study with systematic echocardiography in all patients with streptococcal BSI. Thirdly, all positive BC bottles occurring within 14 days of the first positive BC were considered part of same BSI episode without the possibility to distinguish persistent bacteraemia from several positive BCs obtained in a single initial BC sampling. Fourth, the present study was performed in a specific geographical setting in Scandinavia, which may not represent the prevalence of IE according to streptococcal species in other regions of the world. Finally, it is important to emphasize that the proposed flowchart should not stand alone and the clinical evaluation of the patient is still central in the decision making as stated in the international guidelines [10, 11].

## Conclusion

In addition to the clinical picture, this flowchart based on streptococcal species, number of positive blood culture bottles, and risk factors, can help guide the decision to perform echocardiography in patients with streptococcal bloodstream infections. Since echocardiography results are not available the flowchart should be further validated in a clinical prospective setup with use of systematic echocardiography and in other geographical settings.

## Supplementary Information

**Additional file 1: Table S1.** ICD-, procedure- and ATC-codes.

## Data Availability

All supportive data are available within the article and its supplementary files.

## Abbreviations

AHA: American Heart Association
ATC: Anatomical Therapeutic Chemical
BC: Blood culture
BSI: Bloodstream infection
CHF: Congestive heart failure
CI: Confidence interval
COPD: Chronic obstructive pulmonary disease
DM: Diabetes mellitus
*E. faecalis*: *Enterococcus faecalis*
ESC: European Society of Cardiology
*G. adiacens*: *Granulicatella adiacens*
ICD: International Classification of Diseases
IE: Infective endocarditis
IHD: Ischemic heart disease
NOMESCO: Nordic Medico-Statistical Committee
OR: Odds ratio
PPV: Positive predictive value
*S. aureus*: *Staphylococcus aureus*
SD: Standard deviation
TOE: Transoesophageal echocardiography
TTE: Transthoracic echocardiography
