# Supplementary material for: Proposal for the use of echocardiography in bloodstream infections due to different streptococcal species
Source: BMC Infect Dis. 2021 Jul 16;21:689. doi: 10.1186/s12879-021-06391-2 (PMC8285817; doi:10.1186/s12879-021-06391-2)
Supplement: Supplementary file 1 — Additional file 1: Table S1. ICD-, procedure- and ATC-codes. [file 12879_2021_6391_MOESM1_ESM.docx]

# **Supplementary files**

### **Supplemental Table 1: ICD-, procedure- and ATC-codes**

| Category | Codes | Type |
| --- | --- | --- |
| Cancer | C00 to C97 | ICD-10 |
|  | 140 to 209 | ICD-8 |
| Cardiac implantable electronic device | BFCA0, BFCB0 | Danish procedure codes for treatments |
| Chronic dialysis | BJFD2 | Danish procedure codes for treatments |
| Chronic obstructive pulmonary disease | J42, J44 | ICD-10 |
|  | 490, 491, 492 | ICD-8 |
| Congestive heart failure | I11.0, I51.7, I42.x, I43.x, I50 | ICD-10 |
|  | 427.0, 427.1, 428 | ICD-8 |
| Diabetes Mellitus | A10 | ATC |
| Infective endocarditis | DI33.x, DI38.x, DI39.8 | ICD-10 |
|  | 421 | ICD-8 |
| Ischemic heart disease | I20.x, I23.x, I24.x, I25.x | ICD-10 |
|  | 411, 412, 413, 414 | ICD-8 |
| Native valve disease |  |  |
| Aortic valve disease ^a^ | I35 | ICD-10 |
|  | 395, 396 | ICD-8 |
| Mitral valve disease ^b^ | I34 | ICD-10 |
|  | 394, 396 | ICD-8 |
| Prosthetic valve |  |  |
| Prosthetic mitral valve | KFKD | NOMESCO |
| Prosthetic aortic valve | KFMD | NOMESCO |
| Prosthetic tricuspid valve | KFGE | NOMESCO |
| Prosthetic pulmonary valve | KFJF | NOMESCO |
| Renal disease | I12.x, I13x, N03.x, N04.x, N17.x, N18.x, N19.x, N34.x | ICD-10 |
|  | T858, T859  582, 583, 584, 585, 586, 588 | ICD-8 |
| *ATC, Anatomical Therapeutic Chemical; ICD, International Classification of Diseases; NOMESCO, The Nordic Medico-Statistical Committee*  ^a^ Aortic Stenosis, Aortic Insufficiency, ^b^ Mitral Stenosis, Mitral Insufficiency | | |
